# Symmetry‐breaking in branching epithelia: cells on micro‐patterns under flow challenge the hypothesis of positive feedback by a secreted autocrine inhibitor of motility

**DOI:** 10.1111/joa.12599

**Published:** 2017-03-29

**Authors:** Kimberly C. Martin, Xiaofei Yuan, Gregory Stimac, Kieran Bannerman, Jamie Anderson, Chloe Roy, Fokion Glykofrydis, Huabing Yin, Jamie A. Davies

**Affiliations:** ^1^ Centre for Integrative PhysiologyUniversity of EdinburghGeorge SquareEdinburghEH8 9XBUK; ^2^ School of EngineeringJames Watt BuildingUniversity of GlasgowGL12 8QQUK

**Keywords:** autocrine secretion, branching morphogenesis, cell motility, positive feedback, self‐organisation, shape

## Abstract

Branching morphogenesis of epithelia involves division of cells into leader (tip) and follower (stalk) cells. Published work on cell lines in culture has suggested that symmetry‐breaking takes place via a secreted autocrine inhibitor of motility, the inhibitor accumulating more in concave regions of the culture boundary, slowing advance of cells there, and less in convex areas, allowing advance and a further exaggeration of the concave/convex difference. Here we test this hypothesis using a two‐dimensional culture system that includes strong flow conditions to remove accumulating diffusible secretions. We find that, while motility does indeed follow boundary curvature in this system, flow makes no difference: this challenges the hypothesis of control by a diffusible secreted autocrine inhibitor.

## Introduction

In this report, we test a prediction based on a published hypothesis about epithelial symmetry‐breaking and, by showing that the prediction is not met in a two‐dimensional (2D) system using renal cells, it casts doubt on the universal validity of that hypothesis.

The anatomy of many internal organs is based on the structure of a system of branched epithelial tubes; examples include the airways of the lungs, the urinary collecting ducts of the kidneys, the exocrine ducts of the pancreas, and the tubule systems of the salivary, mammary and uterine glands (for review, see Iber & Menshykau, 2013). The growth of these tubes relies on the cells at the branch tip advancing through the surrounding matrix or mesenchyme, and the creation of new branches depends either on the tip region splitting or, for lateral branching, on a new tip region forming from the side of a stalk. Both mechanisms of new branch formation can be seen in the same developing tree (Watanabe & Costantini, 2004). The differing behaviour of invasive tip cells and non‐invasive stalk cells is a feature of the more general phenomenon of collective cell migration (Khalil & Friedl, 2010). This is characterised by the emergence of specialised ‘leader’ cells, which are characterised by a highly migratory phenotype, and associated with large traction forces exerted against the underlying substrate (Reffay et al. 2014). In contrast, the ‘follower’ cells appear to be dragged passively behind the leader, although it has been shown that they extend protrusions under the cells in front of them, in a sign of active crawling (Farooqui & Fenteany, 2005).

In general, tip and stalk cells develop from the same starting population as when, for example, the lung bud develops from the foregut wall or the ureteric bud develops from the wall of the nephric duct, both probably in response to inductive signals from nearby tissues (Warburton et al. 2010; Woolf & Davies, 2013). Spontaneous branching morphogenesis can also be observed, however, when clones of epithelial cells are grown in simple 2D and three‐dimensional (3D) cultures with no pre‐existing asymmetries, showing self‐organised symmetry‐breaking into tips and non‐tips to be an innate property of these cells (Barros et al. 1995; Sakurai et al. 1997). Recent experimental work with cells in culture has directed increasing attention towards parameters such as cell shape as being a potential factor in directing symmetry‐breaking of leaders and followers (Mark et al. 2010). Cells constrained by culture on 2D scaffolds, as well as in 3D shapes, appear to respond to convex curves with an increasing propensity to protrude, while concave curves in contrast appear to inhibit protrusion.

These responses can be shown to be proportional to the radius of curvature, which has interesting implications in terms of a potential positive feedback loop involved in symmetry‐breaking (through amplification of initial stochastic differences in cell shape) and in collective migration‐based developmental processes (through reinforcing and directing coherent movement of the collectives; Mark et al. 2010; Davies, 2013). It should be stressed that there are theoretical arguments against curvature‐dependent protrusion being the only determinant of branching morphogenesis of tubules such as those in kidney and lung, because it cannot itself promote stable bifurcation (Menshykau et al. 2014): it is being studied by us, and by others, as one aspect of the behaviour of collecting cell migration not as a sole explanation of every aspect.

The curvature‐protrusion feedback model was proposed based on observations of epithelial cells in a culture system that confined them behind a boundary between adhesive and non‐adhesive substrates, but that allowed the non‐adhesive substrate to be removed rapidly to create new and unoccupied territory for the cells to invade. The cells along the released boundary did not advance as a line, but broke symmetry and formed finger‐like shapes with leader cells directing cohorts of followers (Poujade et al. 2007).

Micro‐patterning groups of cells in 3D has led to one hypothesis of the underlying mechanism for the relationship. Nelson and colleagues used a micro‐fabricated mould to create wells of defined geometry in collagen gel, allowing them to generate precisely defined double‐layered tubules consisting of mammary myoepithelial and luminal epithelial cells. When stimulated with appropriate global cytokines, these tubules branched into the surrounding collagen. Shaped wells showed that branching was inhibited at concave regions, and promoted at convex regions (Nelson et al. 2006; see Fig. S4 for a schematic). The authors hypothesized that the position‐dependence of the branch initiation was due to the relative concentration of an autocrine inhibitor secreted by the cells. With secretion rate constant, the greater space available at convex curves would be expected to result in a lower relative concentration, with the converse being true at concave curves. Mathematical simulation supported the idea, and further evidence for this ‘autocrine inhibitor accumulation’ hypothesis came from the use of paired wells, each a rectangle with rounded ends, placed in tandem about 30 μm apart (see Fig. 3 in Nelson et al. 2006; and our Fig. S4 for a summary). In this arrangement, the curvatures of all of the well ends were the same, but the inner ends were predicted to experience more secreted autocrine inhibitor as cells in both the wells contributed to its accumulation in that area. Cells showed much more motility at the outer ends of the wells than the inner ends, suggesting that motility is controlled by a secreted inhibitor rather than directly by curvature. The separation between the wells, about 30 μm apart, was small enough that inhibitor could accumulate but large enough to make direct contact inhibition of locomotion unlikely: images of cells stained for actin in Fig. 2 of the same paper show no evidence of cellular processes capable of spanning such a gap. TGFb1 was suggested as a candidate for the secreted inhibitor, and inhibiting TGFb1 signalling abolished the relationship between well shape and motility. The system did not, though, permit a direct test that differential accumulation (rather than simple presence) of TGFb1 was critical, and it remained formally possible that a different molecule was the regulator that linked well shape to motility.

We have recently shown the secreted autocrine signal, BMP7, to be important in the large‐scale patterning of the renal collecting duct tree. The tips of growing branches avoid colliding with existing branches even if set up in multi‐tree culture conditions that might be expected to cause collisions, and a combination of inhibitor, BMP7‐secreting bead and trans‐filter gradient experiments suggest that this mutual avoidance is due to tips secreting BMP7 and avoiding areas of highest BMP7 intensity: when the signalling system is inhibited branches can run parallel and collide instead of diverging to form a spread tree (Davies et al. 2014). We therefore sought to determine whether this signal was related to leader/follower symmetry‐breaking signals, and developed a 2D micro‐pattern culture system to: (i) verify that renal epithelial cells replicate the relationship between curvature and protrusion observed in other cells; (ii) test that this was due to accumulation of an autocrine inhibitor; and (iii) test whether that inhibitor was the same one, BMP7, implicated in large‐scale patterning of branched renal epithelia.

## Materials and methods

### LifeAct‐MDCK cells

The MDCK II cells used in this study were obtained from the European Collection of Cell Cultures (ECACC; cat. 00062107; lot 10D039) at passage 28. They were modified to constitutively express the live actin‐marker ‘Lifeact‐GFP’ (Riedl et al. 2008). The expression vector pEGFP‐N1‐Lifeact was a kind gift from Dr Tim Czopka at the University of Edinburgh. The cells were cultured at 37 °C and 5% CO_2_ in minimum essential medium Eagle (Sigma, M5650) supplemented with 2 mm L‐glutamine (Gibco, 25030–024), 100 U penicillin/streptomycin (Gibco, 15140) and 5% foetal calf serum (Invitrogen, 10108165).

### Variable‐curvature micro‐patterns

Micro‐patterned substrates, consisting of an amino‐terminated adhesion‐permissive substrate (Sigma‐Aldrich) surrounded by an adhesion‐resistant substrate (PEG‐silane; Gelest), were prepared on glass slides as previously described (Yuan et al. 2015). One day after their last passage, cells were suspended at 2.25 × 10^5 ^cells per mL, and 4 mL of the suspension was placed in the well of a six‐well plate, at the bottom of which was a 1‐cm^2^ glass plate carrying the micro‐patterns, for 2 h at 37 °C. Plates were washed twice with warm phosphate‐buffered saline (PBS), then placed in culture medium.

### Quasi‐Vivo flow culture

Flow experiments were conducted using a Quasi‐Vivo QV500 chamber system (Kirkstall) with a peristaltic pump (EP‐1, Bio‐Rad). Two flow chambers were set up in series, as per the QV500 user manual (Issue Number 3.0), and the volume flow rate through the entire system was measured at 3 mL min^−1^. Prior to placing micro‐patterned cells in the flow chambers, the system was washed with PBS, and then primed with warm complete media. Micro‐patterns were placed facing upwards, narrow end towards the inlet, and flow was maintained for 1.5 h. Control and flow‐treated cells were kept in the same incubator for the duration of the treatment.

### Imaging

Live imaging was conducted using a heated on‐stage chamber, gassed with warmed, humidified air containing 5% CO_2_, on an inverted microscope. For imaging of fixed cells, culture plates were washed briefly with warm foetal calf serum‐free media, then fixed in 4% formaldehyde at room temperature for a minimum of 30 min prior to imaging. Images were captured using a Zeiss AxioImager D1 (inverted) fluorescence microscope equipped with an AxioCam MRm camera and a GFP filter (excitation 470/40; emission 525/50). The 10 × objective allowed the maximum number of patterns to be captured in each image, while still giving sufficient resolution for counting protrusions.

### Image analysis

**Figure 1:**
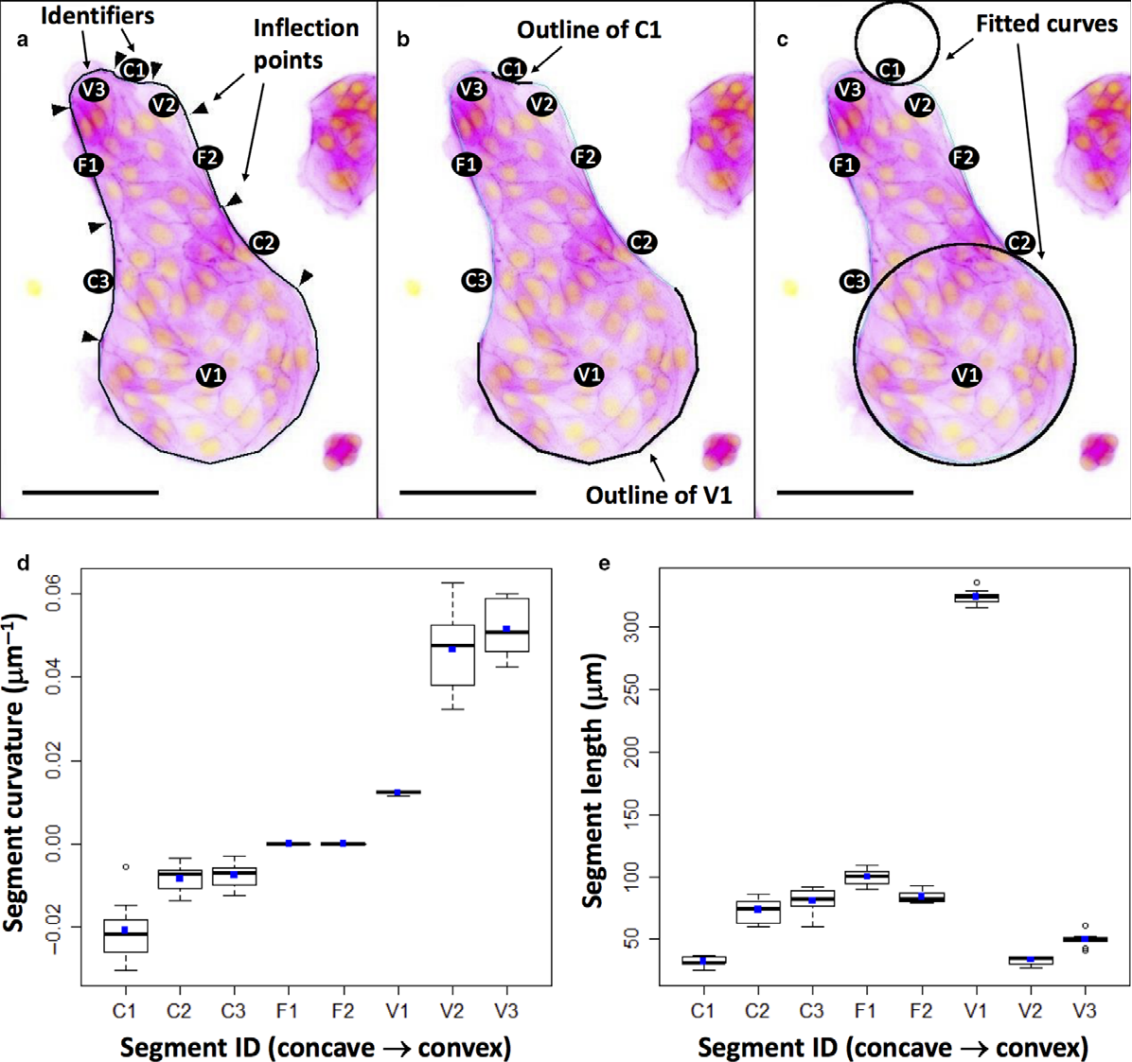
Shape and segmentation of micro‐pattern boundaries. All three images show a typical view of cells growing on one micro‐pattern, the original green‐on‐black fluorescence image being inverted with respect to colours to become magenta on white for ease of labelling. (a) The position of points of inflection (arrowheads) of the curved micro‐pattern periphery (when any temporary lamellipodial extensions cells are making are ignored). The regions between these points of inflection are considered to be segments of the shape and are labelled C1–3 for concave areas, F1–2 for flat and V1 3 for convex. (b) The periphery of just two segments (C1 and V1). (c) Circles fitted to these using ImageJ's ‘Fit Curve’ function: the radii of these circles were used to calculate the curvature of each segment (d). (e) The length of each segment, used in the analyses to normalise protrusion frequency as protrusions‐per‐unit‐length.

The boundary of the micro‐pattern was considered to be divided into distinct segments, defined by points of inflection around the edge of the micro‐pattern, and ImageJ was used to manually delineate each segment, ignoring protrusions (Fig. 1a). Length measurements were taken directly, and then ImageJ's ‘Fit Circle’ function was used to measure the radius of curvature for each segment – defined as the radius of the circle that fits the curve (Fig. 1b,c). Curvature was calculated as radius^−1^, and assigned a negative value for concave curves and a value of 0 for flat sections (equivalent to a circle with an infinite radius). Measurements were made in pixels and converted into μm based on measurement of a scale bar supplied by the microscope software. Segment curvature and length from measurements of 10 independent micro‐patterns are shown in Fig. 1d,e.

For image analysis of samples, segments were included in the analysis if the full length of the segment edge conformed to the expected shape of the micro‐pattern and was visible for assessment for protrusions; for example, segments were ignored when debris or cells that were in the process of being extruded had adhered to cells at the segment edge, potentially concealing protrusions, or if cells had migrated outside the borders of the micro‐pattern. Actin‐containing regions that extended outside the recognisable shape of the variable‐curvature micro‐pattern (beyond the smooth edges defined by the adherent cells) were considered to be protrusions, and where protrusions formed from adjacent cells, they were counted separately.

### Statistical analysis

Test (implemented using the ‘wilcox.test’ function in R) was used to calculate *P*‐values for differences between protrusion rates (protrusion counts normalised by segment length) between segments (Crawley, 2007). Confidence intervals for protrusion rates at each segment were calculated by bootstrapping (sampling with replacement 1000 times), using the R ‘boot’ package. An Exact Poisson rate ratio test (implemented using the ‘rateratio.test’ R package contributed by Michael Fay) was used as an additional test of the significance of relative differences between protrusion rates, and to calculate confidence intervals for protrusion rate ratios. Tables of statistics plotted in the figures here are available as supplementary data (Figs S1 and S2).

### Data archive

Raw images are available on Edinburgh's DataShare server: http://datashare.is.ed.ac.uk/handle/10283/2348.

## Results

### MDCK cells remain confined to patterned substrates but extend lamellipodia dynamically beyond their boundaries

**Figure 2:**
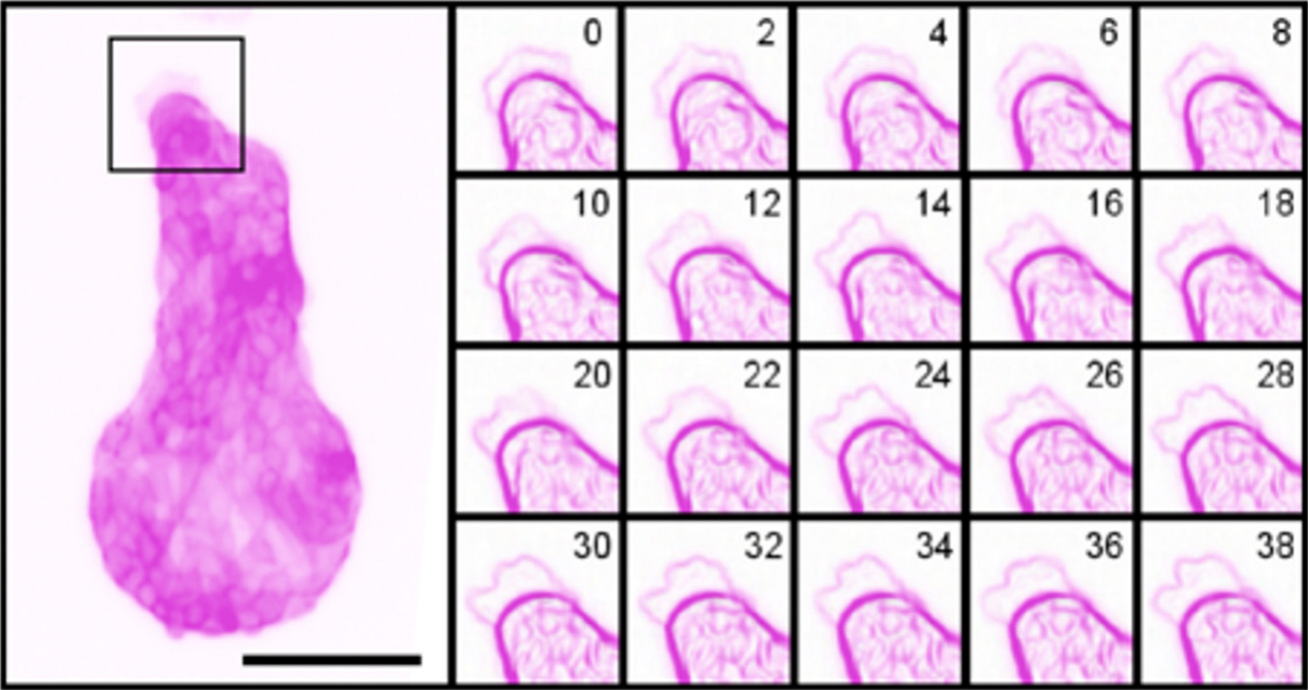
Protrusion dynamics of Lifeact‐MDCK cells on micro‐patterns, again with the colour information inverted to turn green‐on‐black into magenta‐on‐white, for clarity. Images of micro‐patterned cells were captured every 2 min using a 10 × objective, and showed irregular, dynamic protrusions at different regions of the periphery. A time‐series of a 75 μm × 75 μm square (marked in the main panel), using ImageJ's built‐in Edge Detection algorithm to highlight sharp changes in intensity, shows the fluctuations of a typical protrusion. Some protrusions were very stable over time, though they changed shape as shown here, whereas others appeared and disappeared within a handful of frames. Scale bar: 100 μm.

Time‐lapse observation of Lifeact‐MDCK cells, seeded on micro‐patterns, revealed that most cells remained confined to the micro‐pattern but showed dynamic extension of lamellipodia on to the non‐adhesive substrate. A series of images captured every 2 min (Fig. 2) showed actin‐rich processes protruding from the cells. These processes typically had the irregular border and feathery appearance of lamellipodia, which have been observed by previous authors to extend over non‐adhesive surfaces, as well as in the presence of adhesion‐blocking peptides (Bailly et al. 1998). In addition to producing lamellipodia, cells sometimes produced narrow spike‐like and broader‐based triangular protrusions, apparently as transition phases during the establishment and retraction of broad lamellipodia.

**Figure 3:**
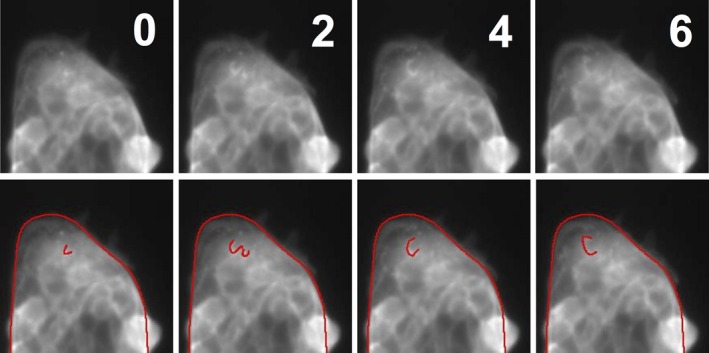
Apical ruffles in live imaged micro‐patterned cells. Images of micro‐patterned Lifeact‐MDCK cells were captured every 2 min using a 20 × objective, and showed evidence of apical surface ruffles driven by actin dynamics. A time‐series of the edge of a micro‐pattern (segments V2, C3 and V3 in a 100 μm × 100 μm square region) is shown, with a typical ruffle highlighted manually in the lower panels.

Live imaging of the micro‐patterned Lifeact‐MDCK also enabled limited observation of actin‐based dynamics on the apical surfaces of cells. This surface showed ruffles, which are folds in the surface of cells associated with compacted actin that move centripetally from the cell periphery and are associated with frustrated adhesion of motile cells (Borm et al. 2005; Giannone et al. 2007; Okeyo et al. 2011). One representative ruffle is highlighted in Fig. 3.

Overall, the behaviour of the cells, in remaining fixed while extending structures associated with motility over their non‐adhesive surroundings, suggested that this system would be suitable for study of the relationship between cell curvature and attempted motility.

### Frequency of protrusion is a function of curvature

In order to test the hypothesis that protrusive activity would be linked to curvature in our system, Lifeact‐MDCK cells were seeded on micro‐patterns, cultured for 24 h and fixed. The frequency of lamellipodial protrusion was determined for each segment of each micro‐pattern. The whole experiment was performed three times, each time including many micro‐patterns, so that each segment was analysed on a minimum of 483 individual micro‐patterns. As a basic statistical check that cells in each segment were behaving independently, the frequency of zero, one, two and more protrusions was compared with a Poisson distribution, and showed a good fit for all segments (Fig. S2). In addition, variance values were approximately equal to the mean as expected for such a distribution (Crawley, 2007).

**Figure 4:**
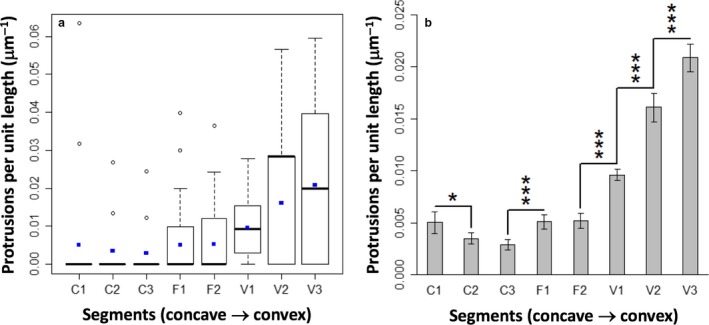
Relationship between curvature and protrusion rate. (a) Tukey box plot of the distribution of protrusion rates by segment. Blue points represent mean values, the bar the median, whiskers extend to outliers up to 1.5 × IQR and further outliers are marked as open spots. (b) Protrusion rates (mean with error bars representing 95% confidence interval) show a strong positive trend with curvature, with significance (*P*‐values calculated using an exact Poisson rate ratio test) indicated by asterisks; *P < 0.05; ***P < 0.001.

When the frequency of protrusions per unit length of segment was plotted against curvature, a strong trend emerged (Fig. 4). Flat segments showed modest protrusion. Segments of increasing convexity showed a marked elevation of the frequency of lamellipodial protrusion (*P *< 0.001). Concave segments, on the other hand, generally showed less protrusion then flat (*P *< 0.001), although the most concave segment C1 was an exception, showing rates of protrusion similar to flat segments. It is clear that, in general, there is a strong relationship between curvature and protrusion, with more convex segments having higher protrusion rates. These results confirm in our system the observations of other researchers (Nelson et al. 2006; Mark et al. 2010) in other systems, and validate the use of our micro‐patterns as a platform for further investigation of the underlying mechanism that couples curvature with motile response.

### The curvature‐motility relationship is unaffected by medium flow

One potential explanation for the relationship between protrusion and curvature, at least in 3D, matrix‐embedded multicellular collectives, involves the secretion of a diffusible autocrine inhibitor of protrusion (Nelson et al. 2006). In this model, cells present at convex curves experience a lower concentration of diffusing inhibitor, while those at concave curves experience a higher concentration as the inhibitor accumulates in zones partially surrounded by cells. While the authors were able to provide some convincing support for their model, and subsequent work has suggested a role for autocrine inhibitor secretion in patterning branching morphogenesis of the renal collecting duct system (Davies et al. 2014), it remained unclear whether this proposed mechanism could explain the relationship between curvature and protrusion seen in a 2D, matrix‐free system. It should be noted that the ratio of protein diffusion constants in free solution and in collagen gels is only about 1.3 : 1 (Galgoczy et al. 2014), so accumulation of secreted proteins does not depend on the presence of a 3D gel.

Whereas Nelson et al. (2006) were able to support the likely role of an autocrine inhibitor by targeting a particular cytokine, TGFb1, in their mammary cell system, TGFb1 has been shown to have a positive rather than negative effect on motility in the MDCK II cell line (Peinado et al. 2003). As an alternative to taking a targeted approach that would involve identifying candidate inhibitors, a blanket approach was taken initially: using rapid flow of culture medium to interfere with the accumulation of any autocrine signalling molecules. This approach has been used to demonstrate perturbation of other aspects of autocrine signalling in cultured cells (Blagovic et al. 2011). Removal of diffusible inhibitor would be expected to have two effects; an overall increase in protrusion (as there is less inhibitor experienced by all cells); and reduction or abolition, if the flushing were perfectly efficient, of the difference between protrusion rates in concave and convex areas.

The use of flow for this purpose depends on choosing a flow rate adequate to disrupt accumulation of secreted molecules but not so strong that cells are subjected to shear stress. Calculations using the formulae of Blagovic et al. (2011), the dimensions of the Kirkstall Quasi‐Vivo perfusion chamber used here, and a flow rate of 3 mL min^−1^ suggests that the effect of flow should massively overwhelm the effects of diffusion on the distribution of molecules (see Fig. S3 for the calculations and Fig. S5 for an empirical demonstration that flow does indeed flush proteins away). The Quasi‐Vivo perfusion culture chamber used here was developed by Ahluwalia and colleagues specifically to minimise shear stress experienced while maximising mass transport (Sbrana & Ahluwalia, 2012). Its use has been successfully demonstrated with ‘difficult’ shear‐sensitive, metabolically demanding primary hepatocytes; maintaining viability and liver‐specific function (Mazzei et al. 2010). At the flow rate of 3 mL min^−1^, the expected shear stress to which cells will be subjected can be calculated, by the equations in Mazzei et al. (2010), to be less than 0.0005 Pa, far less than the shear stress of 0.054 Pa that is known to have only a very limited effect on MDCK cells, even after extended exposure (Wang et al. 2010). The benign nature of this flow was supported by our observing no changes in viability, proliferation rates or morphology of MDCK II cells cultured on patterned substrates under these flow conditions for 12 h: they were indistinguishable from controls.

To test the hypothesis that flow would, by removing accumulated autocrine molecules, abolish the relationship between curvature and motility, MDCK II cells on micro‐patterns were placed in the Quasi‐Vivo flow chamber with the long axis of the pattern parallel to the direction of flow, pointing upstream.

**Figure 5:**
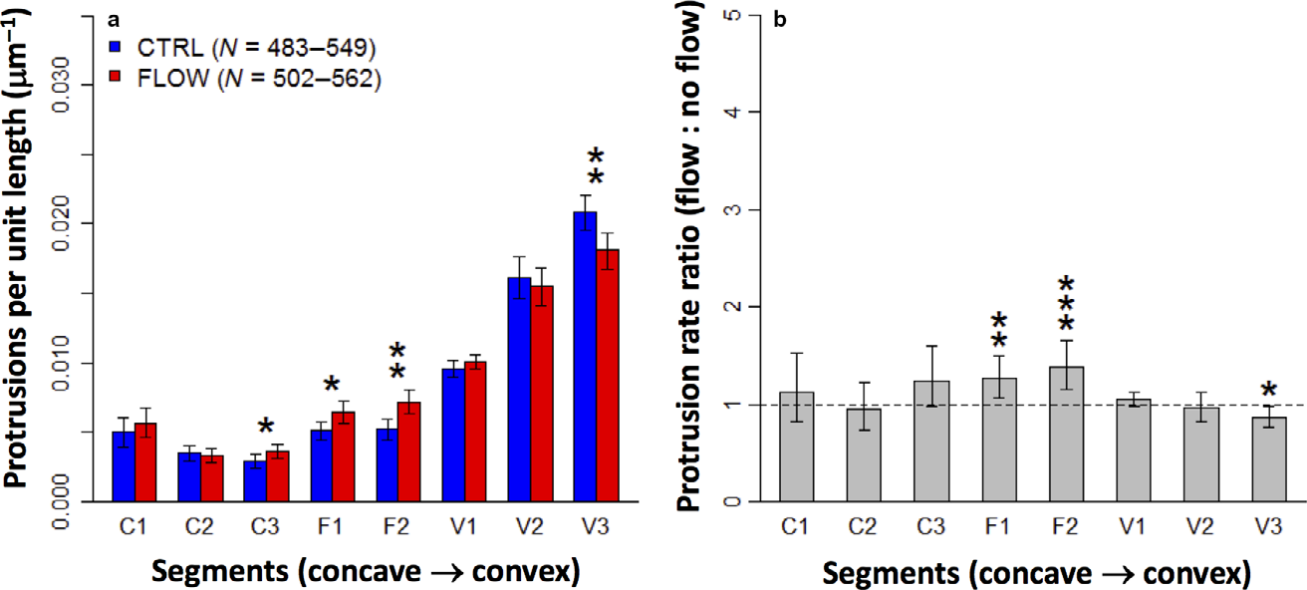
Flow conditions fail to disrupt the relationship between curvature and protrusion. (a) Protrusion rates (mean with error bars depicting 95% confidence interval) show a strong positive trend with curvature, with only minor variations between the control and flow conditions (asterisks indicate significant *P*‐values from Mann–Whitney–Wilcoxon test: *P < 0.05; **P < 0.01). (b) The same data expressed as a ratio between experiment and control, to emphasize the similarity of these sample groups.

Counting protrusions in flow‐treated micro‐pattern segments and plotting protrusion frequency per unit length showed that the relationship between curvature and protrusion was unchanged by exposure to flow (Fig. 5). Concave (C) and flat (F) segments had low protrusion rates, and convex (V) segments showed a positive relationship between curvature and protrusion rate. There was also no general trend of increased protrusion rates at all segments, as would be expected if an inhibitor was usually present and was now washed away by the flow.

Comparing protrusion rates in control and flow‐treated micro‐patterns for each segment did show small differences at some segments. Flow‐treated micro‐patterns showed a slight increase at both flat segments (F1 and F2) and at one of the concave segments (C3), as well as a slight decrease at the most convex segment (V3) (Fig. 5). While the increases at C3, F1 and F2 might suggest that the concentration of an autocrine inhibitor is being reduced, this conclusion is challenged by the lack of significant change at ‘upstream’ segment C1 in particular, as well as by the decrease in the protrusion rate at ‘upstream’ segment V3. It was concluded that the differences in protrusion rates were too small to indicate a meaningful effect of flow treatment.

## Conclusions

Our results demonstrate that MDCK‐II cells show the relationship between curvature and motile activity that can be expected from published observations of other cell types (Nelson et al. 2006; Poujade et al. 2007). Concave boundaries show little protrusive activity while convex ones show more; much more if the convex curvature has a small radius. That pattern would be compatible with the idea of control of curvature by a secreted autocrine inhibitor. If this hypothesis was correct, one would expect that washing the inhibitor away with a strong flow of medium would release all cells from the inhibition, and the relationship between curvature and protrusion would be lost. What is more, one would expect all segments now to show protrusion rates higher than that of the highest rate observed in non‐flowing medium. This did not happen: instead, when the culture system was placed under conditions of strong flow, nothing changed.

The obvious conclusion to be drawn is that the curvature‐protrusion relationship in this 2D system does not in fact rely on secretion and accumulation of a diffusible inhibitor of motility. The robustness of that conclusion depends on the confidence we can have that conditions of flow were adequate to eliminate or at least severely reduce curvature‐controlling accumulations of the secreted molecule. We argue on two grounds that reasonable confidence can be placed in this. The first is the series of calculations made using published equations developed for this exact equipment (Mazzei et al. 2010): our calculations are presented in detail in Fig. S3. They show that the flow rates would be adequate by a very large margin. The second is the quantitative comparison between the behaviour of cells with and without flow. If our calculations were wrong and flow was adequate to remove only some of the inhibitor, leaving enough behind to affect cells, then one would at least expect to see the incidence of protrusion increase, quantitatively, throughout as some inhibitor is lost, even if a relationship between curvature and protrusion was maintained. This did not occur: flow made no difference.

How else might the relationship between curvature and protrusion arise? One possible mechanism is tension‐mediated activation of motility. Tension in the membrane and its associated cytoskeleton will be highest where the membrane is bent around a convex curve and lowest along a concave curve: the correlation between cytoskeletal tension against the substrate, and motility, has been measured directly in MDCK cells on 2D patterned substrates (Rausch et al. 2013). A variety of experiments, in systems as diverse as *Caenorhabditis elegans* sperm, COS1 cells and MDCK cells as used here, has shown that high membrane tension encourages production of a motile process whereas reduced tension inhibits it (Batchelder et al. 2011; Rausch et al. 2013; Tsujita et al. 2015).

Finally, how can the results presented here be reconciled with those of Nelson et al. (2006), who showed the behaviour of mammary cells in tandem‐array 3D wells could be explained by the secreted inhibitor model but not by direct curvature? There are two obvious possibilities. The first is that mechanisms of symmetry‐breaking are organ‐specific rather than universal, and that mammary gland uses a secreted inhibitor whereas the kidney uses direct sensing of curvature. The second possibility is that symmetry‐breaking in 2D systems is very different from 3D because epithelial cells have a different polarity. In 2D culture, the epithelial sheet is discontinuous, ending at the edges of the micro‐pattern, and the basal surfaces of the cells face the substrate rather than the space into which cells might travel. In 3D culture, the epithelium is continuous without free lateral edges, and the basal surface of the cells faces the gel into which cells might travel. It might be that the edge‐effect present in 2D culture allows curvature to exert such a dominant influence that variations in the concentration of a secreted inhibitor are irrelevant to cell behaviour whereas, without edge effects, 3D culture allows the secreted molecule to exert control. These questions could perhaps be explored by using a range of cell types from different organs in both the 2D and 3D systems.

## Author contributions

K.C.M. performed the experiments that produced the data presented here and produced the first draft of the manuscript; G.S., K.B., J.A. and C.R. were project students who performed pilot studies critical to the eventual experimental design; X.Y. produced the micro‐patterns under the supervision of H.Y.; J.A.D. conceived and supervised the study, and contributed to writing the manuscript. No authors have any conflict of interest.

## Supporting information


**Fig. S1.** Data plotted in the graphs in Figs 1, 4 and 5.

**Fig. S2.** Protrusion frequency per segment; *n *= number of segments analysed, μ = mean, σ^2 ^= variance: the predicted Poisson distribution is shown in red. Box plots show cell counts along the edge of each segment (taken from a representative sample of 10 micro‐patterns).

**Fig. S3.** Calculation of flow parameters.

**Fig. S4.** Schematic summary of the results of Nelson et al. (2006).

**Fig. S5.** Empirical demonstration that flow chambers clear soluble proteins effectively.
