# Supplementary material for: Symmetry‐breaking in branching epithelia: cells on micro‐patterns under flow challenge the hypothesis of positive feedback by a secreted autocrine inhibitor of motility
Source: J Anat. 2017 Mar 29;230(6):766–74. doi: 10.1111/joa.12599 (PMC5442143; doi:10.1111/joa.12599)
Supplement: Supplementary file 1 — Fig. S1. Data plotted in the graphs in Figs 1, 4 and 5. [file JOA-230-766-s001.pdf]

Figure S1: data plotted in the graphs in Fig 1., Fig 4 and 5.

Data used in Fig 1:

Micropattern segment curvature measurements (µm^{-1}).

Segment	C1	C2	C3	F1	F2	V1	V2	V3
N	10	10	10	10	10	10	10	10
Mean	-0.021	-0.008	-0.007	0	0	0.012	0.05	0.052
SD	0.007	0.003	0.003	0	0	0.0004	0.01	0.006
Median	-0.022	-0.0073	-0.0070	0	0	0.012	0.048	0.051
IQR	0.007	0.004	0.0037	0	0	0.001	0.014	0.011
Min	-0.030	-0.013	-0.013	0	0	0.012	0.032	0.042
LQ	-0.026	-0.010	-0.0094	0	0	0.012	0.038	0.047
UQ	-0.018	-0.0064	-0.0058	0	0	0.013	0.052	0.057
Max	-0.0054	-0.0035	-0.0028	0	0	0.013	0.063	0.060

Micropattern segment length measurements (µm).

Segment	C1	C2	C3	F1	F2	V1	V2	V3
N	10	10	10	10	10	10	10	10
Mean	32	73	81	100	84	324	33	50
SD	4	9	10	7	5	6	3	5
Median	32	75	82	100	82	324	35	50
IQR	5	14	12	9	6	5	5	3
Min	25	60	60	89	79	315	28	41
LQ	31	66	77	95	81	321	31	49
UQ	36	80	89	104	87	326	36	52
Max	37	86	92	109	93	335	36	61

Data used in Fig. 4:

Protrusion rates for each segment.

Segment	C1	C2	C3	F1	F2	V1	V2	V3
N	516	508	496	507	483	522	532	549
Mean	0.005	0.0035	0.0029	0.0051	0.0052	0.0096	0.016	0.021
SD	0.012	0.0064	0.0057	0.0079	0.0081	0.0064	0.017	0.015
Median	0	0	0	0	0	0.0093	0.028	0.02
IQR	0	0	0	0.01	0.012	0.012	0.028	0.04
Min	0	0	0	0	0	0	0	0
LQ	0	0	0	0	0	0.0031	0	0
UQ	0	0	0	0.01	0.012	0.015	0.028	0.04
Max	0.063	0.027	0.024	0.04	0.036	0.028	0.057	0.06
Mode	0	0	0	0	0	0.012	0	0.02

(please note that the above table is raw data, not normalized for segment length)

Change in protrusion rate with curvature.

Segments	Means diff.	MW p-value	Ratio	95% CIL	95% CIU	RR p-value
C1-C2	-0.0015	0.0380*	0.69	0.52	0.92	0.0121*
C2-C3	-0.0006	0.0112*	0.83	0.64	1.07	0.1645
C3-F1	0.0022	0.0001***	1.76	1.41	2.20	0.0000***
F1-F2	0.0001	0.3106	1.02	0.85	1.23	0.8583
F2-V1	0.0044	0.0000***	1.84	1.59	2.14	0.0000***
V1-V2	0.0064	0.0052**	1.68	1.48	1.90	0.0000***
V2-V3	0.0050	0.0003***	1.29	1.12	1.49	0.0003***

(please note that the above table is from the normalized data)

Data used in Fig. 5:

Protrusion rates under flow conditions.

Segment	Flow	N	Mean	95% CIL	95% CIU	SEM
C1	(-)	516	0.0050	0.0039	0.0060	0.0005
C1	(+)	518	0.0056	0.0046	0.0067	0.0006
C2	(-)	508	0.0035	0.0030	0.0041	0.0003
C2	(+)	517	0.0033	0.0028	0.0038	0.0003
C3	(-)	496	0.0029	0.0024	0.0034	0.0003
C3	(+)	534	0.0036	0.0031	0.0041	0.0003
F1	(-)	507	0.0051	0.0044	0.0058	0.0003
F1	(+)	506	0.0064	0.0057	0.0072	0.0004
F2	(-)	483	0.0052	0.0044	0.0059	0.0004
F2	(+)	502	0.0072	0.0064	0.0081	0.0004
V1	(-)	522	0.0096	0.0090	0.0102	0.0003
V1	(+)	562	0.0101	0.0095	0.0106	0.0003
V2	(-)	532	0.0161	0.0146	0.0176	0.0007
V2	(+)	540	0.0155	0.0141	0.0168	0.0007
V3	(-)	549	0.0209	0.0196	0.0221	0.0006
V3	(+)	562	0.0181	0.0167	0.0194	0.0007

Change in protrusion rates in response to flow conditions.

Segment	Means diff.	MW p-value	Ratio	95% CIL	95% CIU	RR p-value
C1	0.0006	0.4588	1.12	0.82	1.52	0.5115
C2	-0.0002	0.7373	0.95	0.74	1.22	0.7431
C3	0.0007	0.0413*	1.24	0.97	1.59	0.0821
F1	0.0013	0.0178*	1.26	1.07	1.49	0.0059**
F2	0.0020	0.0011**	1.39	1.16	1.66	0.0003***
V1	0.0005	0.2423	1.05	0.98	1.12	0.1466
V2	-0.0007	0.5851	0.96	0.81	1.13	0.6392
V3	-0.0028	0.0033**	0.87	0.77	0.98	0.0200*
