# Supplementary material for: Symmetry‐breaking in branching epithelia: cells on micro‐patterns under flow challenge the hypothesis of positive feedback by a secreted autocrine inhibitor of motility
Source: J Anat. 2017 Mar 29;230(6):766–74. doi: 10.1111/joa.12599 (PMC5442143; doi:10.1111/joa.12599)

Figure S2: Protrusion frequency per segment; N=number of segments analyzed, μ=mean, σ^2=variance: the predicted Poisson distribution is shown in red. Box plots show cell counts along the edge of each segment (taken from a representative sample of 10 micro-patterns)..

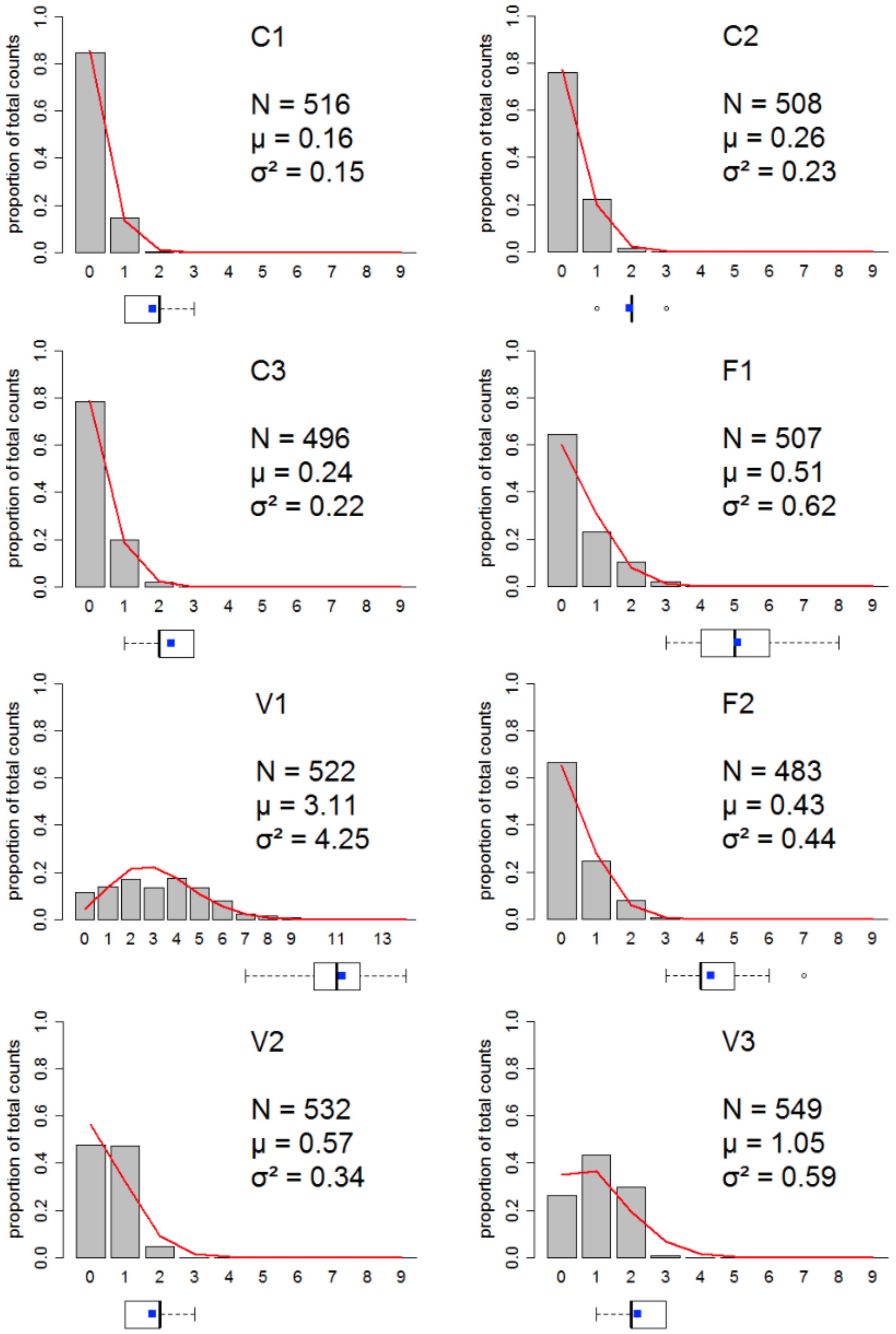

Supplement: Supplementary file 2 — Fig. S2. Protrusion frequency per segment; n = number of segments analysed, μ = mean, σ2 = variance: the predicted Poisson distribution is shown in red. Box plots show cell counts along the edge of each segment (taken from a representative sample of 10 micro‐patterns). [file JOA-230-766-s002.pdf]
