# Supplementary material for: Symmetry‐breaking in branching epithelia: cells on micro‐patterns under flow challenge the hypothesis of positive feedback by a secreted autocrine inhibitor of motility
Source: J Anat. 2017 Mar 29;230(6):766–74. doi: 10.1111/joa.12599 (PMC5442143; doi:10.1111/joa.12599)
Supplement: Supplementary file 3 — Fig. S3. Calculation of flow parameters. [file JOA-230-766-s003.pdf]

# Figure S3: calculation of flow parameters.

The equations we used to estimate flow speed and shear pressure, for the same the culture vessel used here, (including the constants of 2.6 x $10^{-9}$ and 1.3 x $10^{-7}$) were established empirically and published by Mazzei et al. (2010: see p135 in their paper, right column).

**1) Flow speed over the cells:**

The equation for flow speed is: $\text{Flow Speed } (\mathrm{m\,s^{-1}}) = \text{Flow } (\mathrm{\mu L\,min^{-1}}) \text{ x } 2.6 \times 10^{-9} + 1.3 \times 10^{-7}$

At a volumetric flow of 3ml/min (which was the setting of the peristaltic pump, confirmed by weighing fluid delivered in a set time), flow speed over the cells solves to **7.9 µm/s**.

**2) Shear pressure:**

The equation for shear (also determined by Mazzei et al. 2010) is: $\text{Shear (Pa)} = \text{Flow } (\mathrm{\mu L/\,min}) \text{ x } 1.8 \times 10^{-8} + 1.1 \times 10^{-6}$

At this same a volumetric flow of 3ml/min shear pressure solves to **55 µPa**.

The one difference between culture systems is that the cover slip in our system was about 0.84mm thicker than the base used by Mazzei et al. Those authors, however, analysed the effect of raising the base in a similar culture system, and found that a 3mm increase resulted in an approximately 10-fold increase in shear stress. Even with this 10-fold increase in shear, one would expect shear of 0.55 mPa, which is still 100x less than the 54 mPa that was the threshold of effect of shear on MDCK cells established by Wang et al., 2010.

**3) Calculation that flow is far more than adequate to disrupt diffusion-mediated concentration fields:**

These calculations rest on the published work of Blagovic et al. (2011). The critical parameter is the Péclet number, Pe, a dimensionless metric which is the ratio of flow transport to diffusive transport. A value >>1.0 indicates that fluid flow dominates over diffusion. Diffusive transport is represented by the diffusion constant of the molecule, D. The flow term is the product of flow velocity and the characteristic length of the system, for which Blagovic et al. used the height of the chamber (conservative: all other dimensions of the chamber are greater, in their case and in ours), yielding:

$$\mathrm{Pe} = \langle v \rangle\, h / D$$

- $\langle v \rangle$ = 0.0079 mm/s (see section 1 above)
- D = $10^{-6}$ $cm^2$ $s^{-1}$, which is typical of diffusible signalling proteins such as FGF (see Blagovic et al., 2011): this converts to $10^{-4}$ $mm^2$ $s^{-1}$.
- h = 8mm

From these values, Pe ≈ 632. 632 is clearly >> 1.0 so, on this basis, flow will overwhelm diffusion.
