# Supplementary material for: Symmetry‐breaking in branching epithelia: cells on micro‐patterns under flow challenge the hypothesis of positive feedback by a secreted autocrine inhibitor of motility
Source: J Anat. 2017 Mar 29;230(6):766–74. doi: 10.1111/joa.12599 (PMC5442143; doi:10.1111/joa.12599)
Supplement: Supplementary file 4 — Fig. S4. Schematic summary of the results of Nelson et al. (2006). [file JOA-230-766-s004.pdf]

Figure S4: schematic summary of the results of Nelson et al. (2006)

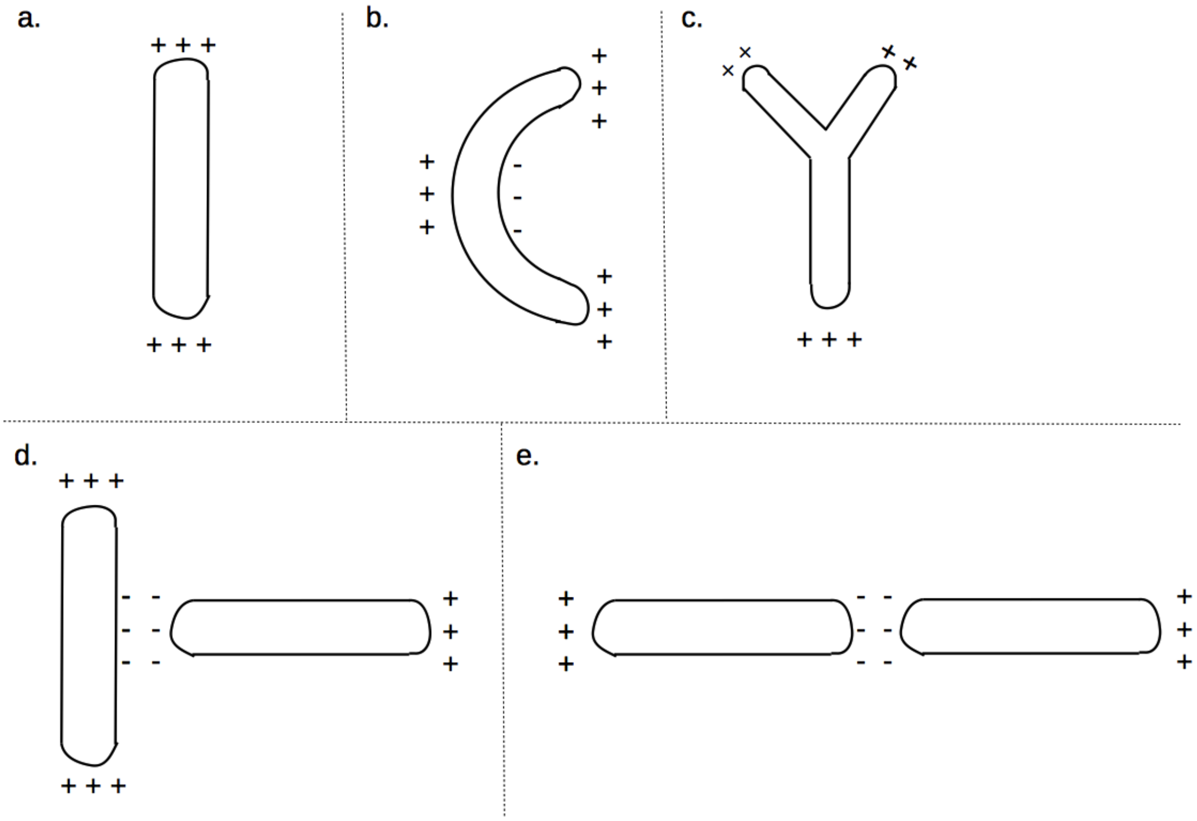

In these diagrams, the well is filled with cells and the surround with a 3D matrix. The plus and minus signs indicate whether rates of cell protrusion/ migration are higher (+) or lower (-) than the rates on the straight edges of the wells.
