# Supplementary material for: Symmetry‐breaking in branching epithelia: cells on micro‐patterns under flow challenge the hypothesis of positive feedback by a secreted autocrine inhibitor of motility
Source: J Anat. 2017 Mar 29;230(6):766–74. doi: 10.1111/joa.12599 (PMC5442143; doi:10.1111/joa.12599)
Supplement: Supplementary file 5 — Fig. S5. Empirical demonstration that flow chambers clear soluble proteins effectively. [file JOA-230-766-s005.pdf]

Figure S5

Empirical demonstration that flow chambers clear soluble proteins effectively.

We outline in the main text a mathematical justification for the assumption that flow over the cells is adequate to clear proteins from the immediate vicinity of the cells, and show detailed calculations in Supplementary Document S3. Here we provide an empirical demonstration.

The demonstration adds two fluorescent tracers to the medium above the cells. One tracer, FITC-IgG (1/100 Sigma F0382), was chosen as a fluorescent protein that is not expected to bind the surface of the MDCK cells. This tracer was used to demonstrate clearance of proteins from the chamber by the flow. The other, Texas-red Wheat Germ Agglutinin (WGA), 20µg/ml, was chosen as a fluorescent protein that binds MDCK cell surface glycoproteins. This tracer was used to demonstrate that proteins can pass freely between the bulk solution and the cell surface and that the cells are not shielded from bulk from by an extracellular matrix. The results are shown below:

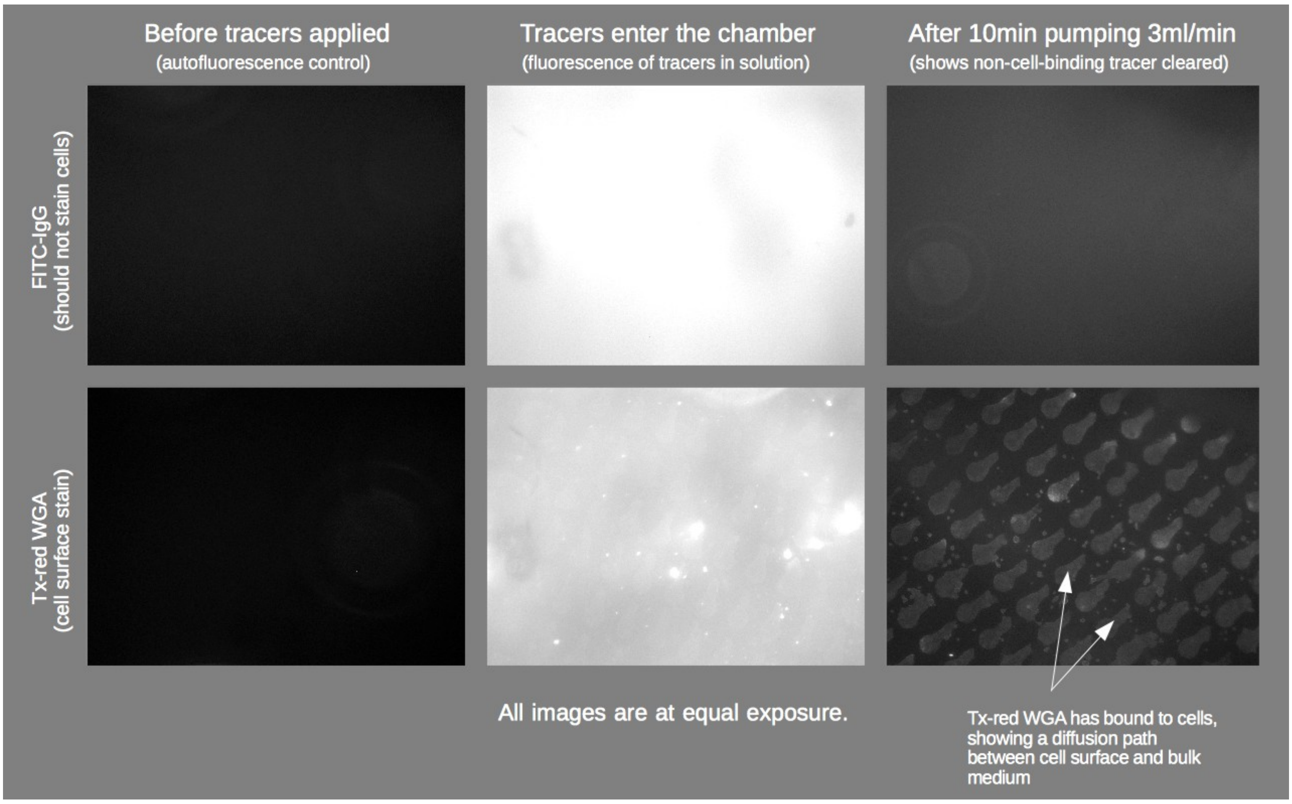

There was no significant autofluorescence before the tracers are added. Images taken with tracer present in the bulk medium showed general bright fluorescent flare, though rows of cell islands could *just* be made out through this background, where WGA was concentrating slightly on them. After 10 mins pumping of plain medium through the flow chamber at the 3ml/min standard rate of

our experiments, the fluorescence signal of the FITC IgG had cleared to bare detectability and there was no evidence of any trapping of the tracer on or around the cell islands. The Tx-red WGA also cleared from the bulk solution but clearly stained MDCK cell surfaces to reveal the rows of cell islands, demonstrating that there was no impediment to movement of proteins between bulk flow and the cell surface.
